# Characterization of human milk oligosaccharides from Chinese mothers and their association with delivery mode and infant eczema

**DOI:** 10.3389/fnut.2026.1804687

**Published:** 2026-05-01

**Authors:** Shan Jiang, Fang Huang, Mickael Hartweg, Mo Chen, Shuxia Wang, Jingyu Yan, Xiangnan Ren, Qiaoji Li, Qu Fu, Irma Silva Zolezzi, Sagar K. Thakkar, Kai Yu, Aristea Binia, Sean Austin, Norbert Sprenger, Yingyao Wang, Zhenyu Yang, Jianqiang Lai, Cathriona Monnard

**Affiliations:** 1 National Institute for Nutrition and Health, Chinese Center for Disease Control and Prevention, Beijing, China; 2 Key Laboratory of Public Health Nutrition, National Health Commission of the Peoples’ Republic of China, Beijing, China; 3 Key Laboratory of Human Breast Milk Science, Chinese Center for Disease Control and Prevention, Beijing, China; 4 Nestlé Institute of Health Sciences, Nestlé Research, Beijing, China; 5 Clinical Research Unit, Nestlé Research, Lausanne, Switzerland; 6 Key Laboratory of Separation Science for Analytical Chemistry, Dalian Institute of Chemical Physics, Chinese Academy of Sciences, Dalian, China; 7 Clinical Research Unit, Nestlé Research, Beijing, China; 8 Nestlé Health Science, Taizhou, China; 9 Nestlé Product Technology Center, Vevey, Switzerland; 10 Nestlé Institute of Health Sciences, Nestlé Research, Lausanne, Switzerland; 11 Nestlé Institute of Food Safety and Analytical Sciences, Nestlé Research, Lausanne, Switzerland; 12 CNS Academy of Nutrition and Health (Beijing Zhongyinghui Nutrition and Health Research Institute), Beijing, China

**Keywords:** C-section, human milk oligosaccharides, infant allergy, infant eczema, maternal, mode of delivery

## Abstract

**Objective:**

This study investigated how HMO profiles in Chinese mothers’ milk are affected by lactation stage and delivery mode (C-section vs. vaginal).

**Study design:**

The study was based on a cross-sectional multi-center human milk study in China. Twenty-four HMOs were quantified using UPLC/MS/MS, and statistical tools were used to identify profiles and clusters of HMOs. The relationship between HMO concentrations, delivery mode and infant eczema was explored at each lactation stage.

**Results:**

The study included 635 mother-infant pairs. The concentrations of HMOs varied according to lactation stage and five distinct HMO clusters were observed based on significant associations between HMOs. Milk from mothers who delivered by C-section had significantly lower concentrations of 2’FL, LNnT, 3’SL, LNFP-III, DFLNHa, DSLNT, LNDFH-II, LNnDFH-II, and DFpLNnH compared to vaginal delivery. The odds ratio for eczema was reduced in infants whose mothers had higher concentrations of LSTb in mature milk (>90 days).

**Conclusion:**

HMOs in Chinese human milk vary based on lactation stage and cluster based on their structure. C-section delivery lowers the concentrations of several HMOs in the milk compared to vaginal delivery. Higher LSTb inversely associated with parent-reported infant eczema. Further research is required to confirm these findings and to understand the underlying mechanisms.

## 1 Introduction

Human milk (HM) is the optimal and recommended sole source of nutrition for the first 6 months of life (1, 2). Breastfeeding is associated with numerous health benefits, including a lower risk of infectious diseases, improved cognitive performance and long-term protection against non-communicable diseases (2). Beyond nutrients, HM acts as a conduit between mother and infant. While macronutrients support infant growth (3), bioactive factors like human milk oligosaccharides (HMOs) have been proposed to play a role in other aspects of infant health (4).

HMOs are non-digestible carbohydrates abundant in HM (5). Research into the role of HMOs over the past decade has begun to elucidate their role in supporting infant health. HMOs are a well-known substrate for beneficial bacteria in the infant gastrointestinal tract and are capable of modulating the microbiome (6). Linked to their effects in the gastrointestinal tract and on the microbiome, HMOs are purported to play a role in supporting immune tolerance (5). Recent evidence indicates they may play a role in allergy prevention (7–11), as well as in reducing the risk of respiratory and rotavirus infections (12–15).

The protective effect of HMOs may in part be linked to their concentrations and profiles in the milk, which have been shown to vary over time and in response to maternal and environmental factors. The HMO profile of HM is determined by the genetic status of the mother (16), and is influenced by lactation stage, gestational age, mode of delivery, maternal nutritional status, and geographic location (4). There is a noteworthy variation in HMO composition across lactation stages (17), which appears to be conserved across different geographical locations (18, 19). Major differences in HMO profiles reflect genetic differences in histo-blood group antigen expression (7, 16, 20). Numerous previous studies have measured HMO composition in milk from lactating mothers across different regions of China (21–29), yet few studies have explored how maternal factors such as giving birth by cesarean section (C-section), affect HMO composition and how HMO composition associates with infant health outcomes (29–32).

C-section delivery, in particular, is one maternal factor that has repeatedly been shown to impact the composition of bioactives in HM (17, 30, 33–37). While the precise mechanism is not completely understood, it is hypothesized that C-section may affect maternal physiology and / or trigger inflammatory processes that affect the secretion of milk bioactives (37, 38). Certain HMOs have been reported to be lower in the milk of mothers who delivered by C-section (2’FL, 3’SL, LNFP-II, LNFP-III, LNnDFH), which also aligns with reports of lower sialic acid in C-section milk (37). In contrast, HMOs such as LNT and 6’SL are reported to be increased in milk from mothers who delivered via C-section (17, 30, 32). Two previous studies have investigated the link between C-section, HMOs and infant allergy, although with inconsistent findings. Chen et al. (32) found higher 2’FL in milk from mothers who delivered via C-section and noted that infants who developed eczema consumed breastmilk with higher 2’FL concentrations. In contrast, Sprenger et al. (8) found a trend toward lower risk of IgE associated eczema when C-section-born infants received breastmilk containing FUT2-dependent milk oligosaccharides.

Given the limited number of studies and inconsistencies in the existing literature, the objective of the current study was to explore the impact of mode of delivery (C-section vs. vaginal) on HMO abundance in the milk. Additionally, we sought to understand if mode of delivery and HMO composition affect infant health outcomes.

## 2 Methods

### 2.1 Study design

The current study utilized samples collected from November 2011 to June 2013 within the Chinese Center for Disease Control and Prevention’s cross-sectional multi-center human milk study, detailed in Yin and Yang (39). Human milk samples were collected cross-sectionally across lactation stages from different lactating mothers according to the following definitions: Colostrum, 0–7 days; Transitional milk, 8–21 days; Mature milk 1, 22–50 days; Mature milk 2, 51–90 days; Mature milk 3, 91–340 days. Only mothers of Han ethnicity who delivered at full term (>37 weeks gestation) were included.

This study utilized a combination of two datasets for a larger sample size: an initial set of 437 samples and a later set of 198 samples, totaling 635 subjects from eight provinces and municipalities. Laboratory analyses for the two datasets were completed in 2018 and 2023, respectively. To account for potential batch effects, a dataset variable was included in all analyses.

### 2.2 Sample and data collection

Detailed information on the milk sampling and data collection can be found in the original publication (39). Briefly, human milk samples were collected at the second morning feed (9:00–11:00 a.m.) using an electric breast pump. Mothers were instructed to empty a single full breast, and an aliquot of 50 mL was used for the characterization of milk composition. The remaining milk was returned to the mother for feeding the infant. Each sample was aliquoted into 15-mL freezing tubes, labeled, and stored at −80 °C until analysis. Retrospective information on infant eczema was collected via parental questionnaire, which was completed during the study visits - “Has your child ever had the following allergic conditions? (1) No, (2) Eczema, (3) Asthma, (4) Atopic dermatitis, (5) Rhinitis, (6) Others, (7) Unknown.” Data on diarrhea and respiratory illness (colds, bronchitis, pneumonia, etc.) were collected during the visit, focusing on occurrence in the past 2 weeks. The study procedures involved one visit where trained staff administered standardized questionnaires. These included one for lactating women, collecting demographic information, socio-economic status, lifestyle and medical history, and another for breastfeeding infants, gathering data on birth outcomes, breastfeeding status, and dietary intake.

### 2.3 Ethical and legal considerations

Written informed consent was obtained from all subjects. The study was conducted according to the guidelines laid out in the Declaration of Helsinki. The original cross sectional multi-center human milk study was approved by the Ethics Committee of the former National Institute of Nutrition and Food Safety of Chinese Center for Disease Control and Prevention (39). The current study was approved by the Ethical Committee of the National Institute of Nutrition and Health of Chinese Center for Disease Control and Prevention (No. 2022-010).

### 2.4 HMO analysis

HMOs mentioned in this publication are shown alongside their structures in Supplementary Table S1. Transitions for 24 HMOs were followed by Ultra performance liquid chromatography (UPLC) (Acquity, Waters, Milford, MA, United States) coupled to mass spectrometry (MS) (Xevo TQ-XS, Waters) following the method of Li et al. (22) with minor modifications. Briefly, samples were defrosted at room temperature, then placed in an ultrasonic bath (5–10 min, 35 °C). The sample was mixed well, and an aliquot (50 μL) was mixed with water (700 μL) before centrifugation (7,160 × *g*, 6 min, 4 °C). An aliquot (100 μL) of the aqueous layer was diluted with ethanol (200 μL) and the mixture was centrifuged (7,160 × *g*, 6 min, 4 °C). An aliquot (50 μL) of the supernatant was diluted with acetonitrile/water (1/1, 150 μL) and transferred to a vial suitable for the instrument autosampler.

HMOs were separated by UPLC on an Acquity BEH Amide column (130 Å, 1.7 μm, 2.1 × 150 mm, Waters) using a gradient of water in acetonitrile and ammonium acetate (Supplementary Table S2). Electrospray ionization mass spectrometry (MS) was performed in the negative ion mode and oligosaccharides were detected using a multiple reaction monitoring experiment, with desolvation gas at 800 L/h, cone gas at 150 L/h, nebulizer gas pressure at 6.3 bar, capillary voltage at 2.2 kV, cone voltage at 45 V, and capillary temperature at 600 °C. The isobaric pairs of 3’-SLNFP-II and 6’-SLNFP-VI and of LNDFH-I and LNnDFH-I co-eluted, thus only 20 HMOs could be quantified. The HMOs were quantified against an external calibration curve of HMOs which were previously isolated from human milk (22). The specific fragmentation, precursor-to-product ion pair(s) and the collision energy used for each oligosaccharide are listed in Supplementary Table S3.

### 2.5 Milk group classification

Maternal genetic polymorphisms affecting FUT2 and FUT3 enzyme activity result in four major milk groups with distinct HMO profile. Milk samples were categorized into one of the four milk groups depending on the levels of 2′FL and LNFP-II present in the samples (40). Samples were assigned to milk group 1 if 2′FL levels were greater than 25 mg/L and LNFP-II levels were greater than 35 mg/L. Samples were assigned to milk group 2 if LNFP-II levels were above 35 mg/L and 2’FL levels were below 25 mg/L. Samples were assigned to milk group 3 if 2’FL levels were greater than 25 mg/L and LNFP-II levels were below 35 mg/L. Samples were assigned to milk group 4 if 2’FL levels were below 25 mg/L and LNFP-II levels were below 35 mg/L.

### 2.6 Statistical analysis

The data analysis focused on 20 independent HMOs available in both datasets. Baseline characteristics were summarized as means ± SD, medians (IQR), or frequencies (%). HMO levels below the lower limit of quantification (LLoQ) were assigned a value of 0.5 × LLoQ. Graphical representations of HMO levels included boxplots for lactation stages and provinces, a Spearman correlation plot for HMOs, and a dendrogram from agglomerative clustering to show hierarchical relationships. Nonparametric tests were applied to skewed logarithmically transformed data. Categorical variables were analyzed using chi-square tests.

The primary analysis focus was to investigate the association between delivery mode and HMO concentrations both overall and by lactation stage based on biological rationale and prior evidence. Multiple linear regression analyses investigated the association between HMO concentrations and delivery modes, adjusting for lactational stage, maternal allergy history, parity, and gestational diabetes mellitus (GDM) history, along with interaction terms involving delivery modes. Logistic regression explored associations between HMO levels and infant health outcomes listed in the design section, adjusting for confounders such as lactational stage, maternal allergy history (specifically for eczema), and interactions terms with lactational stages. Elastic net regression was used as a robustness check. Differences in HMO concentrations are reported as estimated difference in least square means between groups, with respective 95% confidence intervals (CIs). The association between infant health outcomes and HMO concentrations is presented as odds ratios (OR) with 95% CIs.

Statistical analyses were performed using R version 4.5.2 (R Foundation for Statistical Computing, Vienna, Austria) and SAS version 9.4 (SAS Institute Inc., Cary, NC, United States), with significance set at *p* < 0.05. No overall multiplicity control was applied due to the exploratory nature and sample size. The risk of false discoveries was mitigated by evaluating significance, effect size, and biological plausibility, along with consistency across analyses and alignment with findings from other studies.

## 3 Results

### 3.1 Study participants

A total of 692 mother infant pairs were screened for this study. Fifty-seven were subsequently excluded with 43 being excluded due to preterm birth and 14 for not being of Han ethnicity. The remaining 635 mother infant pairs were used for the current analysis. Of these, 298 mothers delivered their infant vaginally and 337 gave birth via C-section. Details of the recruitment can be found in the flow diagram in Supplementary Figure S1.

### 3.2 Demographic characteristics of mothers and their infants

The characteristics of mothers and their infants are summarized in Table 1. Mothers had a median age of 26.5 years old. All mothers were healthy entering pregnancy and 68.1% had a healthy pre-pregnancy BMI. For the majority it was their first live birth (79.1%). Additionally, 3.2% of mothers reported being diagnosed with GDM during pregnancy, and 12.1% reported a history of allergy.

**Table 1:** Maternal and infant characteristics.

Maternal characteristics	Vaginal birth	C-section	Total *N* (%)	*p* value
Age, years (min, max)	26.2 (23.1, 29.3) (*n* = 298)	27.3 (24.6, 29.8) (*n* = 335)	26.5 (23.6, 29.7) (*n* = 633)	0.019
Province and municipalities				<0.001
Heilongjiang	41 (22.2%)	144 (77.8%)	185 (29.1%)	
Gansu	66 (86.8%)	10 (13.2%)	76 (12%)	
Beijing	41 (50.0%)	41 (50.0%)	82 (12.9%)	
Shandong	40 (58.8%)	28 (41.2%)	68 (10.7%)	
Shanghai	14 (43.7%)	18 (56.3%)	32 (5.0%)	
Zhejiang	6 (22.2%)	21 (77.8%)	27 (4.3%)	
Guangdong	42 (47.7%)	46 (52.3%)	88 (13.9%)	
Yunnan	48 (62.3%)	29 (37.7%)	77 (12.1%)	
Pre-pregnancy BMI				0.091
Underweight	48 (20.3%)	43 (16.0%)	91 (18.0%)	
Normal	163 (69.1%)	181 (67.3%)	344 (68.1%)	
Overweight and obesity	25 (10.6%)	45 (16.7%)	70 (13.9%)	
Parity				0.432
1	229 (77.6%)	266 (80.4%)	495 (79.1%)	
2	66 (22.4%)	65 (19.6%)	131 (20.9%)	
Allergy history				0.619
Yes	32 (11.3%)	42 (12.8%)	74 (12.1%)	
No	251 (88.7%)	285 (87.2%)	536 (87.9%)	
GDM				0.172
No	290 (98%)	323 (95.8%)	613 (96.8%)	
Yes	6 (2%)	14 (4.2%)	20 (3.2%)	

Infant characteristics	Delivered vaginally	C-section	Total *N* (%)	*p* value
Lactation stages				0.059
Colostrum (1–7 d)	97 (32.6%)	81 (24%)	178 (28%)	
Transitional (8–21 d)	116 (38.9%)	128 (38%)	244 (38.4%)	
Mature 1 (22–50 d)	24 (8.1%)	32 (9.5%)	56 (8.8%)	
Mature 2 (51–90 d)	15 (5%)	19 (5.6%)	34 (5.4%)	
Mature 3 (91–340 d)	46 (15.4%)	77 (22.9%)	123 (19.4%)	
Infant sex				0.873
Male	162 (54.4%)	186 (55.2%)	348 (54.8%)	
Female	136 (45.6%)	151 (44.8%)	287 (45.2%)	
Respiratory diseases in previous 2 weeks (cold, bronchitis, pneumonia etc.)				0.869
No	277 (93.6%)	314 (94%)	591 (93.8%)	
Yes	19 (6.4%)	20 (6%)	39 (6.2%)	
Diarrhea in previous 2 weeks				0.317
No	276 (92.9%)	319 (94.9%)	595 (94.0%)	
Yes	21 (7.1%)	17 (5.1%)	38 (6.0%)	
Eczema since birth				0.270
No	271 (92.2%)	297 (89.5%)	568 (90.7%)	
Yes	23 (7.8%)	35 (10.5%)	58 (9.3%)	

Over half of infants were male (54.8%). In terms of illness, reports of respiratory illness (colds, bronchitis, pneumonia, etc.) and diarrhea were low for the majority (93.8 and 94.0% no reports, respectively). Prevalence of parent-reported eczema since birth was 9.3%.

Mothers delivering via C-section were slightly older (27.3 vs. 26.2 years; *p* = 0.019). The province and municipalities they resided in also differed. Aside from these differences, there were no other significant differences between mothers having given birth vaginally or by C-section, or differences between infants having been delivered by either method.

### 3.3 HMO profiles in Chinese mothers’ milk

**Figure 1:**
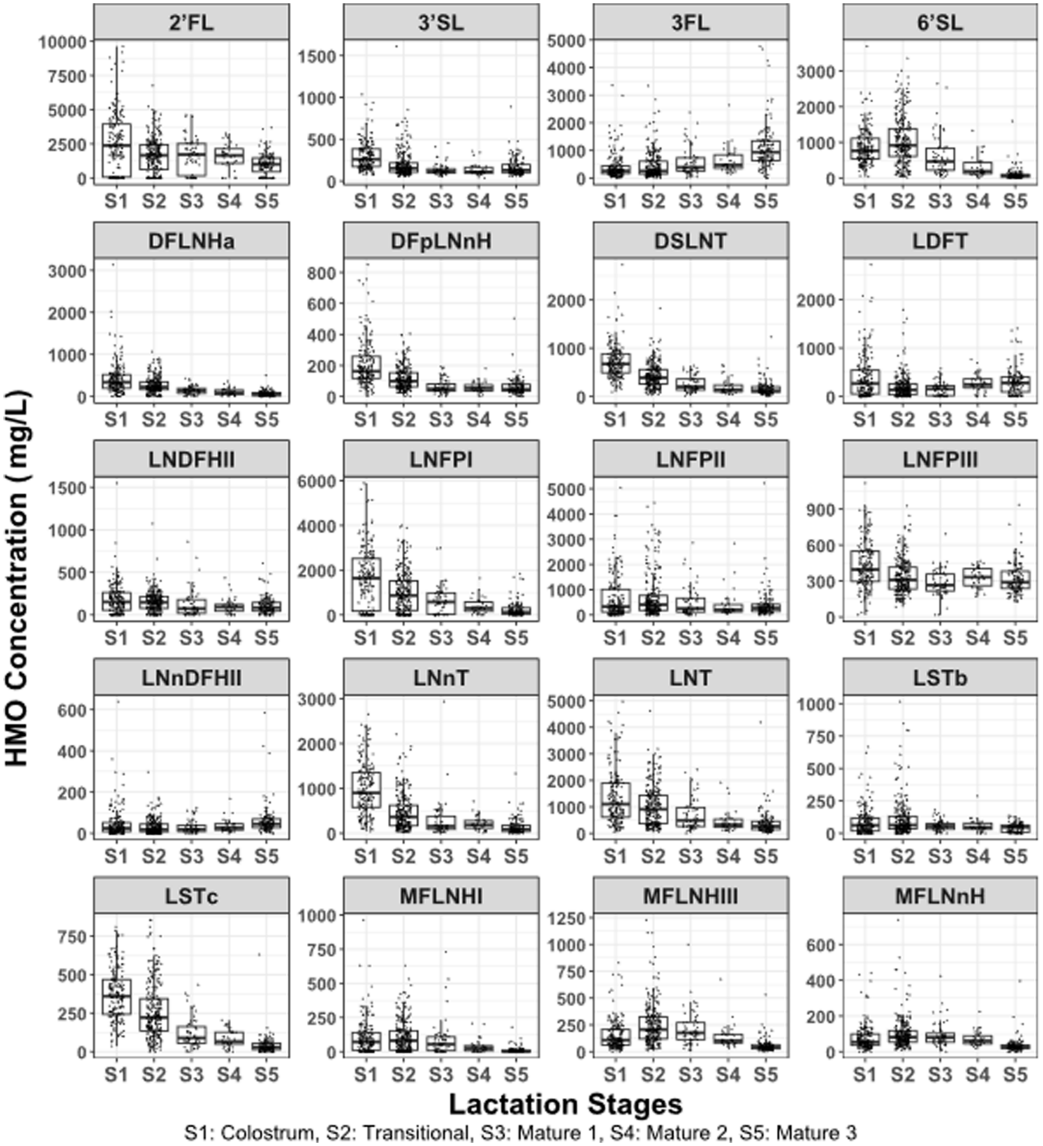
HMO concentrations across lactation stages. Lactation stage was defined as colostrum 0–7 days; transitional 8–21 days, mature 1 22–50 days, mature 2 51–90 days, mature 3 >90 days. Data from *n* = 635 mothers across 8 provinces and municipalities in China.

The concentration of each HMO across lactation stage is shown in Figure 1. The concentration of most of the HMOs was higher at early stages of lactation and decreased as lactation progressed. A notable exception to this was 3FL, which increased from 261 mg/L (162, 436.5 mg/L) in colostrum to 936 mg/L (630, 1349.6 mg/L) in mature 3 milk (*p* < 0.001).

The concentration of each HMO according to mother’s resident province and municipality can be found in Supplementary Figure S2. The sample distribution across lactation stages varies among the eight provinces and municipalities (Supplementary Table S4), particularly between Heilongjiang and Gansu, as well as between Shandong and Gansu. Heilongjiang had fewer colostrum samples (18.9% vs. 36.8% in Gansu and 28.0% overall) and thus, showed a more mature milk profile (Mature 3 milk: 25.9% vs. 13.2% in Gansu and. 19.4% overall). Similarly, Shandong demonstrated a more mature milk profile (Mature 1 milk: 20.6% vs. 5.3% in Gansu and 8.8% overall).

**Figure 2:**
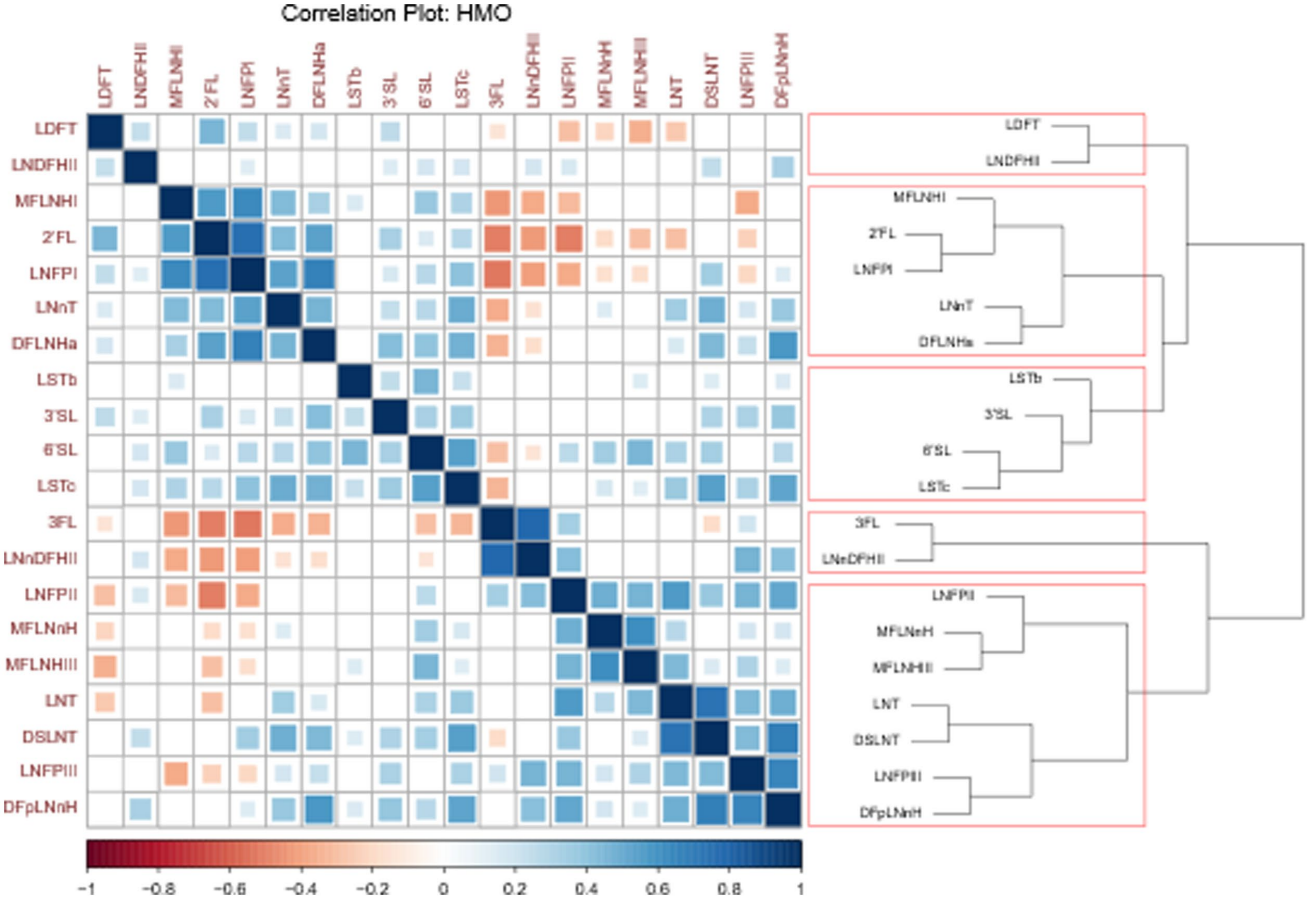
Correlations of measured HMOs based on significant associations. Spearman correlation values range from −1 (dark red) to 1 (dark blue). Size and color of squares is proportional to the correlation coefficient value. The dendrogram on the right is divided into five HMO clusters, delineated by red boxes, with HMOs within the same cluster grouped together within each box. The HMOs in the correlation matrix were reordered based on the clustering results.

Significant correlations were observed between several HMOs, and five HMO clusters were identified (Figure 2). Cluster 1 included LDFT and LNDFHII (difucosylated HMOs). Cluster 2 included MFLNH-I, 2’FL, LNFP-I, LNnT and DFLNHa, with all except LNnT containing an α-1,2-linked fucose residue, thus only found in significant concentrations in milk of secretor mothers. Cluster 3 included LSTb, 3’SL, 6’SL and LSTc (monosialylated HMOs). Cluster 4 included 3FL and LNnDFHII (α-1,3-linked fucoses). Finally, cluster 5 included LNFPII, MFLNnH, MFLNHIII, LNT, DSLNT, LNFPIII and DFpLNnH, mainly comprising HMOs with α-1,3-linked fucose to GlcNAc, as well as LNFP-II which contains an α-1,4-linked fucose, and LNT and DSLNT, which share the common galactose—GlcNAc—galactose core, consistent with other members of this cluster.

### 3.4 HMO differences by milk groups

Mothers were categorized into one of four milk groups based on the presence or absence of specific α-1,2 and α-1,3/4- fucosylated HMOs as proxies for the activity of fucosyltransferases 2 (FUT2) and 3 (FUT3) (Supplementary Figure S3). LDFT and LNDFH-II were both highest in group 1 mothers’ milk. 2’FL, 3’SL, LNnT, LNFP-I, MFLNH-I and DFLNHa were all highest in group 3 mothers. 3FL, LNFP-II, LNnDFH-II, MFLNnH and DFpLNnH were highest in group 2 mothers and 6’SL, LNT, LNFP-III, DSLNT, LSTb, LSTc and MFLNH-III were all highest in group 4 mothers.

### 3.5 Impact of delivery mode on HMO profiles

The impact of delivery mode (vaginal vs. C-section birth) on HMO concentrations across lactation stages was explored, while adjusting for parity, maternal allergy, GDM, dataset indicator and interaction terms. Analysis revealed that the associations between delivery mode and HMO concentrations were primarily evident in colostrum and transitional stage (despite a non-significant interaction term delivery mode * lactation stages) (Table 2).

**Table 2:** Differences* in HMO concentrations (mg/L) by delivery mode and lactation stage.

HMO	C-section vs vaginal	C-section	Vaginal	Lactation stage	*p*-value
2’FL	−581.13 (−1084.47, −77.8)	2416.77	2997.90	Colostrum	0.024
LNnT	−146.5 (−272.38, −20.62)	686.66	833.16	Colostrum	0.023
LNFP-III	−65.05 (−115.34, −14.76)	391.68	456.73	Colostrum	0.012
LNnDFH-II	−18.96 (−37.69, −0.24)	51.50	70.46	Colostrum	0.048
DFLNHa	−100.28 (−173.66, −26.9)	305.28	405.56	Colostrum	0.008
DFpLNnH	−52.59 (−83.92, −21.26)	181.72	234.32	Colostrum	0.001
3’SL	−45.54 (−85.93, −5.15)	214.36	259.91	Transitional	0.028
DSLNT	−105.34 (−178.47, −32.22)	347.99	453.33	Transitional	0.005
LNDFH-II	−88.08 (−167.48, −8.67)	126.90	214.98	Mature 1	0.030
LNFP-III	−39.02 (−75.25, −2.79)	324.69	363.71	Avg across lactation	0.035

*The estimated difference between delivery mode at each lactation stage has been adjusted by parity, maternal allergy, GDM, dataset indicator, and interaction terms with delivery modes. Only HMOs with significant differences between delivery models for each lactation stage have been included.

By individual lactation stage, mothers who gave birth via C-section had lower concentrations of 2’FL, LNnT, LNFP-III, DFLNHa, LNnDFH-II and DFpLNnH in colostrum, lower concentrations of 3’SL and DSLNT in transitional milk, and lower concentrations of LNDFH-II in mature milk. Across all lactation stages, mothers who delivered via C-section had lower average concentrations of LNFP-III compared to mothers who delivered vaginally.

### 3.6 HMO associations with reported eczema, diarrhea and respiratory illness

The association between HMOs and eczema was investigated by adjusting for lactation stage, maternal allergy history and interaction term between HMO and lactation stages. Prevalence of eczema by lactation stage can be visualized in the scatter plot (Supplementary Figure S4). Parent-reported eczema was recorded as occurring anytime since birth (distribution of reported eczema cases across lactation stages is shown in Supplementary Table S5). In the mature 3 lactation stage (>90 days), an inverse association between LSTb concentration and eczema was observed (OR 0.978, *p* = 0.007; 95% CI: 0.963, 0.994). Specifically, for each 1 mg/L increase in LSTb concentration in mature milk beyond 3 months, the odds of eczema decreased by 2% (Supplementary Table S5). Further analysis incorporating an interaction term between HMO and maternal allergy reinforced this association in Supplementary Table S5. The impact of delivery mode on eczema was also investigated, but no significant association was found (data not shown).

Data were also collected on parent-reported diarrhea and respiratory illness (colds, bronchitis, pneumonia, etc.) as incidences of the illness during the 2 weeks prior to the visit, except for mothers enrolled during colostrum (0–7 days) and transitional milk (8–21 days) collection for whom the period was shorter (7 and 13 days, respectively). No significant associations were observed between measured HMOs and either diarrhea or respiratory illness (data not shown).

## 4 Discussion

HM is the gold standard for infant nutrition and a biologically active system that supports growth and development in early life (1, 2, 41, 42). Beyond its nutritional components, HM contains a myriad of bioactives—including HMOs—that contribute to infant development (43). Influential predictors of HMO composition and concentration include maternal genetics, lactation stage, gestational age, mode of delivery, maternal nutritional status, and geographic location (4). We found that the profile of HMOs varied across lactation stages, with the majority decreasing in concentration over time, the exception being 3FL, which increased as lactation progressed. These findings align with those of global systematic reviews on HMO concentrations (44), other individual European studies (17), as well as other findings from Asia (45) and Chinese mother-infant cohorts (21, 22, 25, 29).

We investigated the association between HMOs and the mode of infant delivery. Interestingly, we found lower concentrations of LNFP-III across all lactation stages in the milk of mothers who delivered by C-section compared with those who delivered vaginally. Within each lactation stage, specific differences were observed in the HMO composition of milk including, for example, lower 2’FL, LNnT, LNFP-III, LNnDFH-II, DFLNHa, and DFpLNnH in colostrum, lower 3’SL and DSLNT in transitional milk, lower LNDFH-II in mature milk. Lower levels of 2’FL, 3’SL (17, 32) and 6’SL (30) have previously been observed in the colostrum of European and Chinese (Xi’an city) mothers who delivered by C-section compared with those who delivered vaginally.

The precise mechanisms underlying the observed effect of C-section delivery on milk composition are not understood. One hypothesis could involve inflammatory processes: C-section delivery is associated with greater low-grade inflammation, as indicated by higher circulating C-reactive protein (CRP) post-delivery (46). Previous studies have shown an association between maternal conditions linked to elevated CRP (such as overweight) and lower HM carbohydrate (lactose) content (47). In ruminants, inflammatory conditions such as mastitis result in decreased lactose content of the milk (48), as a result of immune activation, which shifts glucose utilization away from lactose synthesis and toward meeting the energy requirements of the activated immune system (49). It could be proposed that a similar mechanism operates in humans and C-section-induced inflammation may result in a reduction in the building blocks for HMO synthesis. The increases in CRP associated with C-section delivery were identified mostly in early lactation and tend to resolve thereafter, which may explain the predominant impact of C-section on HMO composition of the early milk identified in this study. Another possibility is that the hormonal differences induced by C-section compared to vaginal delivery (oxytocin, cortisol, prolactin) may affect the glycosylation machinery necessary for HMO synthesis. Future studies should take into account factors such as labor induction—which is known to impact hormone secretion patterns (50)—as well as whether the C-section was elective or emergency, to help further our understanding of the mechanisms underlying the effect of C-section on the HMO composition of HM.

The current study associated HMO composition with parent-reported outcomes—principally eczema. Higher LSTb concentrations were inversely associated with eczema in both vaginal and C-section born infants, although the effect size was not large. This effect was observed primarily in mature milk, which likely relates to the greater prevalence of eczema during the mature milk feeding period in our cohort (27%) compared with earlier timepoints (1.4%). Existing evidence on the protective effect of human milk and its bioactives in childhood allergy is not conclusive (11). Some studies suggest a protective effect of HMOs on allergy risk (8–10, 46), while others have shown either no significant effect (51, 52) or increased risk (32, 53). Of the protective associations reported, 2’FL and FUT-dependent HMOs were associated with reduced risk of IgE-eczema at 2 years of age (8); while 6’SL, DSLNT, LNFP I, and LNFP-III were associated with lower risk of cow’s milk protein allergy within the first 18 months of life (9).

The protective effects of HMOs on allergy risk may be confounded by infant age and hereditary atopy risk. The associations between LSTb and infant eczema in our study was observed in mothers with (*p* = 0.05) and without (*p* = 0.02) a history of allergy. Maternal allergy has been proposed to impact HMOs in the milk and Al-Kaabawi et al. (54) found higher 2’FL, LNFP I, LNT, 3’SL, 6’SL, LSTb, DSLNT, sialylated, neutral, and total HMO concentrations in mature milk of non- allergic mothers compared to allergic mothers. Our cohort was mostly nonatopic (87.9%) and thus, we are unable to explore the relationship between maternal atopy, HMO profiles and eczema. Nonetheless, our finding of varying associations in both atopic and nonatopic mothers may indicate that factors other than maternal atopy are influencing HMO profiles and infant eczema. It would be important for future studies to capture data on infant genetics, since the association of HMOs with allergy may also depend on infant risk status, as was previously shown for C-section born infants (8) and infants with a high genetic risk score (7). Notably, alpha-1,2 fucosylated HMOs like 2’FL, LNFP-I and DFLNHa were observed to possibly have a protective effect on recurrent wheezing in infants with high genetic risk score (7).

The current study enrolled mothers from 8 provinces and municipalities across China. Heilongjiang had fewer colostrum samples and thus, showed a more mature HMO profile compared to other provinces that had a greater number of samples from the earlier lactation stages, which likely affected the HMO concentration and thus, the observed differences between provinces. We are not the first to report regional differences in HMOs: Liu et al. (29) found significant differences in the sum of the 6 measured HMO concentrations (2′FL, 3-FL, LNT, LNnT, 3′SL and 6′SL) across 6 regions in China (Changchun, Lanzhou, Chengdu, Tianjin, Guangzhou, Shanghai) in a population of mostly (>90%) Han ethnicity. While genetic factors may have had an influence, it is also possible that environmental factors such as maternal diet or climate may play a role (29). Additional evidence from studies applying similar sampling and timing, as well as measures of maternal dietary intake, will help to better understand the influence of local environmental factors on differences in HMO profiles across geographies.

The variability in HMO concentrations reported across different populations (44) may in some cases be linked to the geographical distribution of milk groups (55, 56). In the current study, we explored differences in the concentrations of HMOs between four defined groups. The cut offs of 25 mg/L 2′FL and 35 mg/L LNFP-II were used in our study to determine secretor status. The majority (72%) of the mothers in the current cohort presented the secretor phenotype (active FUT2 enzyme), which is similar to what has been reported previously for other Chinese cohorts (30, 57, 58) and fits within the global prevalence values for secretor phenotype of between 64 and 89% (26). LNFP-II was used to determine the status of the other polymorphic fucosyltransferase FUT3. 3FL and LNFP-II show the highest concentrations in milk group 2 and show little presence in milk groups 3 and 4. This is somewhat similar to existing findings where 3FL was still present in group 3 and 4 (17). We identified the highest concentrations of 2’FL, LNnT, LNFP-I and DFLNHa in group 3 milk when FUT2 is active and FUT3 is inactive, as reported previously (16, 17). Similar to previous European studies, we identified LNT, LNFP-III, DSLNT, LSTb and MFLNH-III at higher concentrations when both FUT2 and FUT3 are inactive (milk group 4) (17, 59). In our data, 3’SL and 6’SL seem to be less affected by milk groups, confirming previous reports (17).

Clustering analysis was performed based on the correlations between the measured HMOs over time in mothers’ milk and identified 5 clusters of correlated HMOs. Cluster 1 consisted of two HMOs LDFT and LNDFH-II, both of which are difucoslyated. This specific cluster has not been reported in previous studies, however, LDFT and LNDFH-I have been found to cluster previously (16). Unfortunately, LNDFH-I was not part of the quantified HMOs in the current study. The second cluster we identified contained 2’FL, LNnT, LNFP-I, DFLNHa and MFLNH-I, which—with the exception of LNnT—all contain an α-1,2-linked fucose residue, which are thus only found in significant concentrations in the milk of secretor mothers. Although LNnT is not fucosylated, previous studies (17, 60) show that LNnT tends to be at higher concentrations in the milk of secretor mothers. MFLNH-I was not measured in previous studies reporting HMO cluster analyses. However, 2’FL, LNFP-I DFLNHa and LNnT were previously shown to cluster together (7, 16, 61). Cluster 3 in our study consisted of 6’SL, 3’SL, LSTb and LSTc, all of which are monosialylated HMOs. Both 6’SL and LSTc have previously been shown to cluster together (7, 16). Cluster 4 in our study contained 3FL and LNnDFH-II, which contain only α-1,3-linked fucoses. Both HMOs have previously been reported to cluster together, alongside LNFP-II, -III and -V (16). The final cluster consisted of LNT, DSLNT, LNFP-II, LNFP-III, MFLNnH, MFLNH-III and DFpLNnH. This cluster predominantly contains the HMOs containing α-1,3-linked fucose, the exceptions being LNFP-II - which contains an α-1,4-linked fucose - and LNT and DSLNT. Previous studies (17) have shown that MFLNH-III, DSLNT, LNT and LNFP-III tend to be higher in group 4 milks in which neither FUT2 nor FUT3 are active, and LNFP-II is higher in group 3 milk in which FUT2 is inactive. Neither MFLNH-III nor DFpLNnH were included in the previous study (17) but based on their structural features they could also tend to be higher in group 4 milk in which FUT2 and FUT3 are inactive. In summary, HMOs tend to cluster based on their structure and also potentially maternal genetics. Future studies are warranted to understand the potential functional implications of these clusters in HM.

### 4.1 Strengths and limitations

This is the first study to explore the association of the 20 HMOs measured with C-section birth and infant eczema—from a large study covering a wide geographical region of China. However, some limitations should be noted. Although the impact of delivery mode on HMO concentrations during lactation stages is supported by prior evidence, the lack of a significant interaction term suggests that these differences need further confirmation in future studies. The impact of other maternal factors such as pre-pregnancy BMI on HMO concentrations was not examined due to missing data regarding pre-pregnancy BMI for 20% of mothers. Additionally, many of the associations were observed in colostrum, a period of substantial variability in HMO concentrations. Future studies with adequate longitudinal sampling across lactation are warranted to verify our findings. It is also important to note that eczema was parent-reported, which may have led to a misclassification of true eczema. Nonetheless, caregiver-reported measures were previously shown to demonstrate sufficient validity for the epidemiological study of eczema (62). The study did not apply false discovery rate control for the association results, which may result in findings that should be interpreted cautiously and validated with additional evidence. We also lacked information on maternal and infant genetics, as well as the infant microbiome. Future studies should include measures of blood specific IgE to validate our findings and provide a more holistic understanding of the role of HMOs in allergy prevention.

## 5 Conclusion

We identified lower concentrations of HMOs in the milk of Chinese mothers who delivered via C-section compared to those who delivered vaginally. The precise mechanisms underlying these observations should be investigated in future studies. We observed five distinct HMO clusters in the milk of Chinese mothers, which were based on structure, but potentially also maternal genetics. Lastly, LSTb was found to be associated with reduced risk of parent-reported eczema in infants. A more integrative approach - incorporating maternal and infant genetics, infant microbiome and environmental factors—may help to confirm existing evidence on how maternal factors shape HMO concentrations in milk and further shed light on the role of HMOs in supporting immune development in early life.

## Data Availability

The datasets generated during and/or analyzed during the current study are available from the author on reasonable request. Requests to access the datasets should be directed to yangzy@ninh.chinacdc.cn.
