# Supplementary material for: Characterization of human milk oligosaccharides from Chinese mothers and their association with delivery mode and infant eczema
Source: Front Nutr. 2026 May 1;13:1804687. doi: 10.3389/fnut.2026.1804687 (PMC13178246; doi:10.3389/fnut.2026.1804687)
Supplement: Supplementary file 1 [file Data_sheet_1.pdf]

Supplementary Tables

Supplementary Table 1 HMOs mentioned in this publication and their structures using symbol nomenclature

HMO Abbreviation	HMO structure
2'-FL	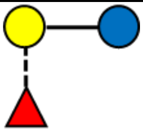
3-FL	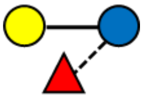
3'-SL	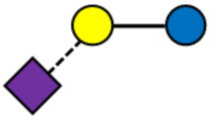
6'-SL	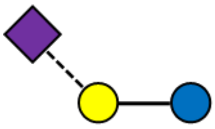
LDFT	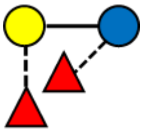
DSLNT	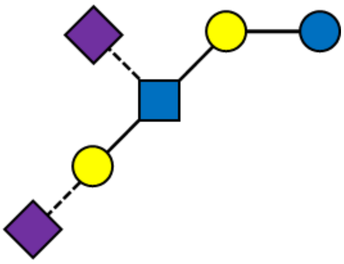
LNT	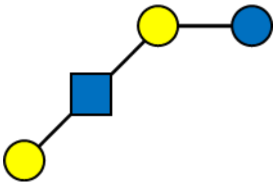
LNnT	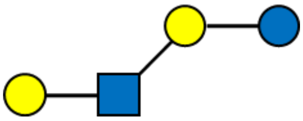
LNFP-I	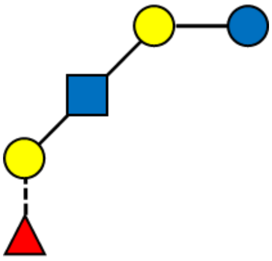
LNFP-III	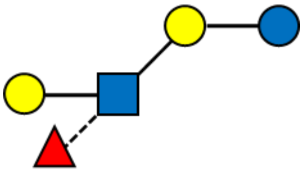

LNFP-II

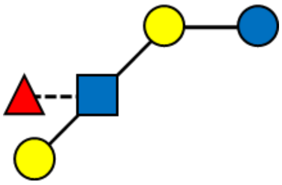

LSTb

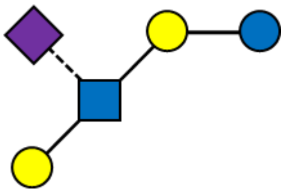

LSTc

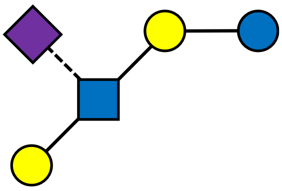

LNnDFH-II

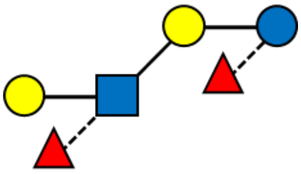

LNDFH-II

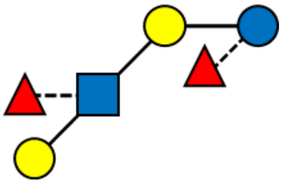

LNDFH-I

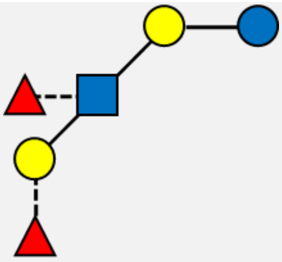

LNnDFH-I

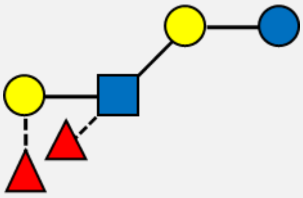

3’’’-SLNFPII

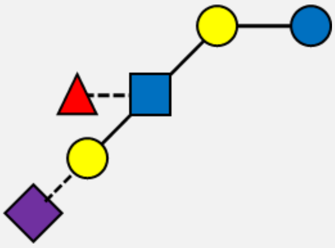

6’’’-SLNFPVI

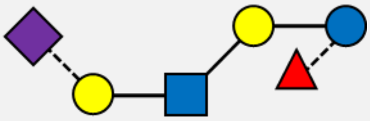

MFLNnH

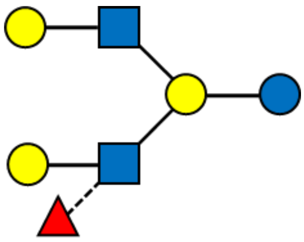

MFLNH-III

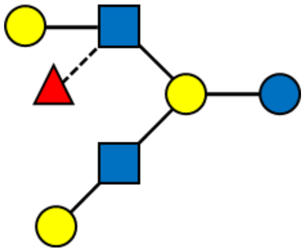

MFLNH-I

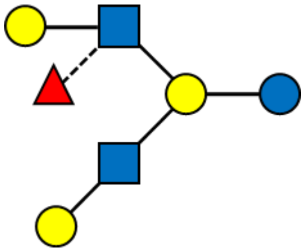

DFpLNnH

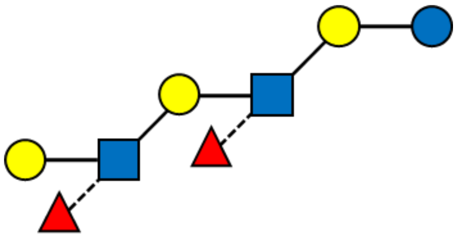

DFLNHa

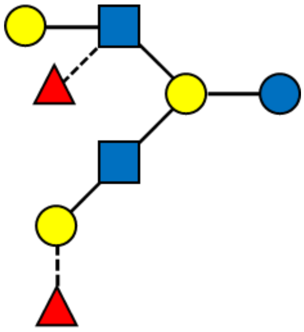

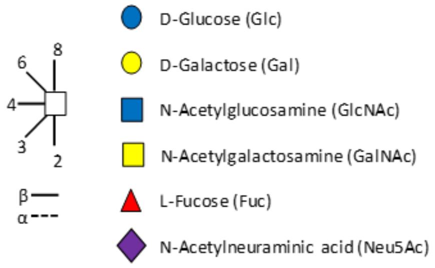

Supplementary Table 2 UPLC Gradient for separation of HMO

Time (min)	Flow (mL/min)	Acetonitrile (%)	Water (%)	Ammonium acetate (50 mM) (%)
0.0	0.3	71	19	10
2.0	0.3	71	19	10
9.5	0.3	46	44	10
10.1	0.3	20	70	10
10.5	0.3	20	70	10
11.6	0.3	71	19	10
15.0	0.3	71	19	10

Supplementary Table 3 Multiple Reaction Monitoring Conditions for Mass Spectrometer

HMO	Retention Time (min)	Primary ion pair		Secondary ion pair	
		m/z	CID energy (eV)	m/z	CID energy (eV)
2'-FL	4.9	487 → 325	8		
3-FL	5.2	523 → 179	20	487 → 323	20
3'-SL	4.4	632 → 290	28		
6'-SL	5.0	632 → 290	32		
LDFT	6.1	633 → 325	13		
DSLNT	6.5	644 → 290	28		
LNT	6.3	706 → 202	20		
LNnT	6.4	706 → 263	20		
LNFP-I	7.0	852 → 325	22		
LNFP-III	7.2	852 → 364	20		
LNFP-II	7.3	852 → 348	20		
LSTb	6.4	997 → 290	45		
LSTc	6.5	997 → 290	45		
LNnDFH-II	8.1	998 → 364	26		
LNDFH-II	8.1	998 → 348	26		
LNDFH-I	8.1	998 → 325	35	998 → 348	20
LNnDFH-I	7.7	998 → 325	20		
3'-SLNFPII	6.8	1143 → 290	50	1143 → 348	48
6'-SLNFPVI	7.2	1143 → 290	48		
MFLNnH	8.4	1217 → 526	25	1217 → 364	25
MFLNH-III	8.3	1217 → 672	30	1217 → 202	43
MFLNH-I	8.2	1217 → 526	28		
DFLNHa	8.7	1363 → 672	28	1363 → 364	45
DFpLNnH	8.9	1363 → 875	28	1363 → 364	45

Supplementary Table 4 Distribution of lactation stages for each province and municipality

Provinces	Colostrum	Transition	Mature 1	Mature 2	Mature 3	Total
Heilongjiang	18.9%	37.8%	9.7%	7.6%	25.9%	185
Gansu	36.8%	43.4%	5.3%	1.3%	13.2%	76
Beijing	32.9%	42.7%	2.4%	3.7%	18.3%	82
Shandong	19.1%	32.4%	20.6%	4.4%	23.5%	68
Shanghai	31.3%	40.6%	3.1%	3.1%	21.9%	32
Zhejiang	33.3%	48.1%	11.1%	0.0%	7.4%	27
Guangdong	33.0%	29.5%	11.4%	9.1%	17.0%	88
Yunnan	35.1%	41.6%	5.2%	5.2%	13.0%	77
total	28.0%	38.4%	8.8%	5.4%	19.4%	635

Values are the percentage of milk samples in each lactation stage coming from each province and municipality.

Supplementary Table 5 HMO association with parent-reported eczema since birth

Lactation stage	Reported eczema cases†	Median (IQR) LSTb concentration (mg/L)	Odds ratio (OR)*	P-value
Early stage **	417 (Yes=6, 1.4%)	63.0 (33.1-132.5)	1.001 (0.995, 1.007)	0.840
Mature 1 Stage	55 (Yes=11, 20.0%)	54.5 (30.0-76.5)	1.012 (0.996, 1.027)	0.139
Mature 2 Stage	34 (Yes=9, 26.5%)	45.8 (31.1-78.8)	0.985 (0.960, 1.010)	0.226
Mature 3 Stage	111 (Yes=32, 28.8%)	46.9 (9.0-63.0)	0.978 (0.963, 0.994)	0.007
Mature 3 Stage (without maternal allergy history)	89 (Yes=22, 24.7%)	50.1 (18.5-68.3)	0.979 (0.963, 0.995)	0.020
Mature 3 Stage (with maternal allergy history)	22 (Yes=10, 45.5%)	29.3 (9.0-47.7)	0.975 (0.950,1.000)	0.051

*The model is adjusted for lactation stage, maternal allergy, dataset indicator, and interaction terms (HMO * lactation stage, HMO * maternal allergy). The odds ratio (OR) is estimated based on a 1 mg/L increase in HMO concentration.

† Parents answered the question "Has your child ever had the following allergic conditions? ① No ② Eczema ③ Asthma ④ Atopic dermatitis ⑤ Rhinitis ⑥ Others ⑦ Unknown". Values in the table represents number of subjects with counts and percentages of subjects reporting eczema

**Early stage: These two lactation stages colostrum & transitional are combined as 'early stage' due to low number of eczema cases reported in the colostrum stage

The descriptive median (IQR) HMO concentrations are presented.

Supplementary Figures

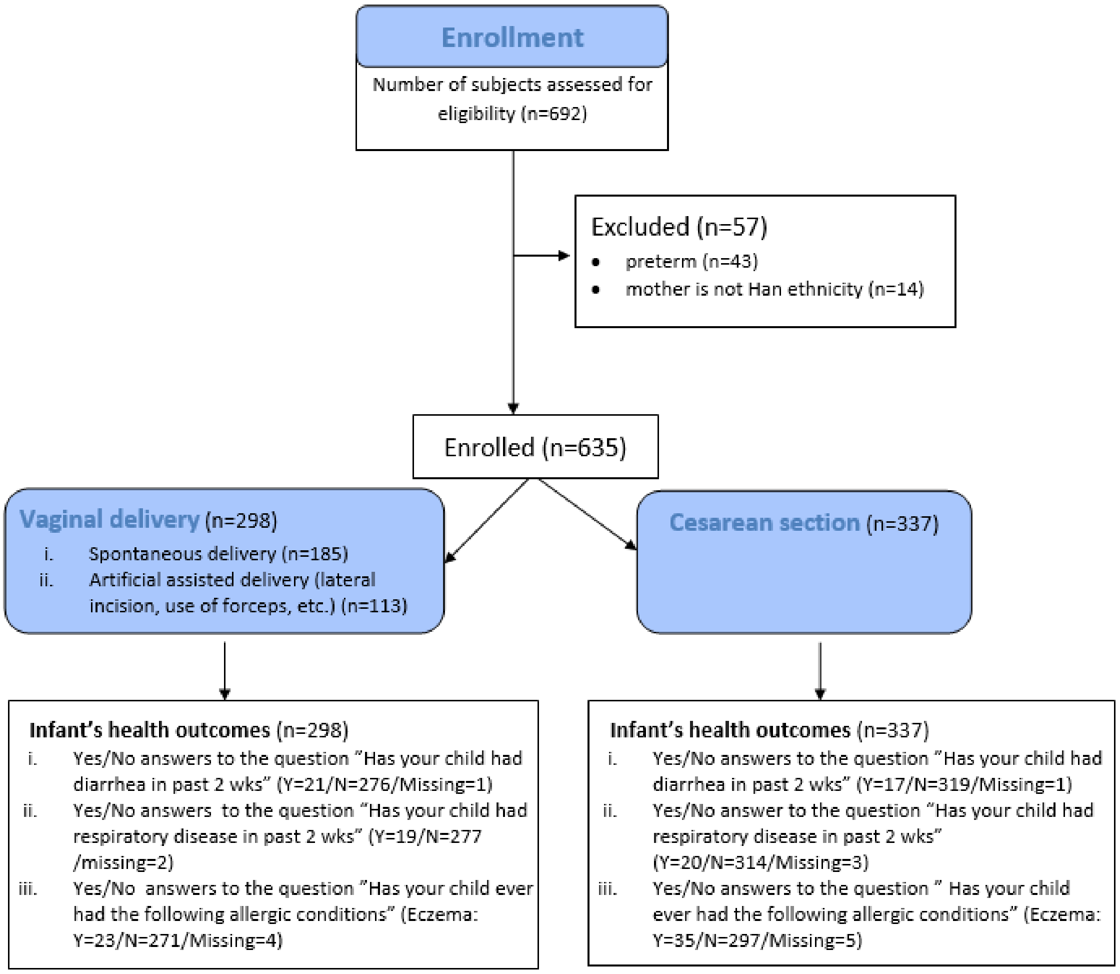

Supplementary Figure 1 Study flow diagram

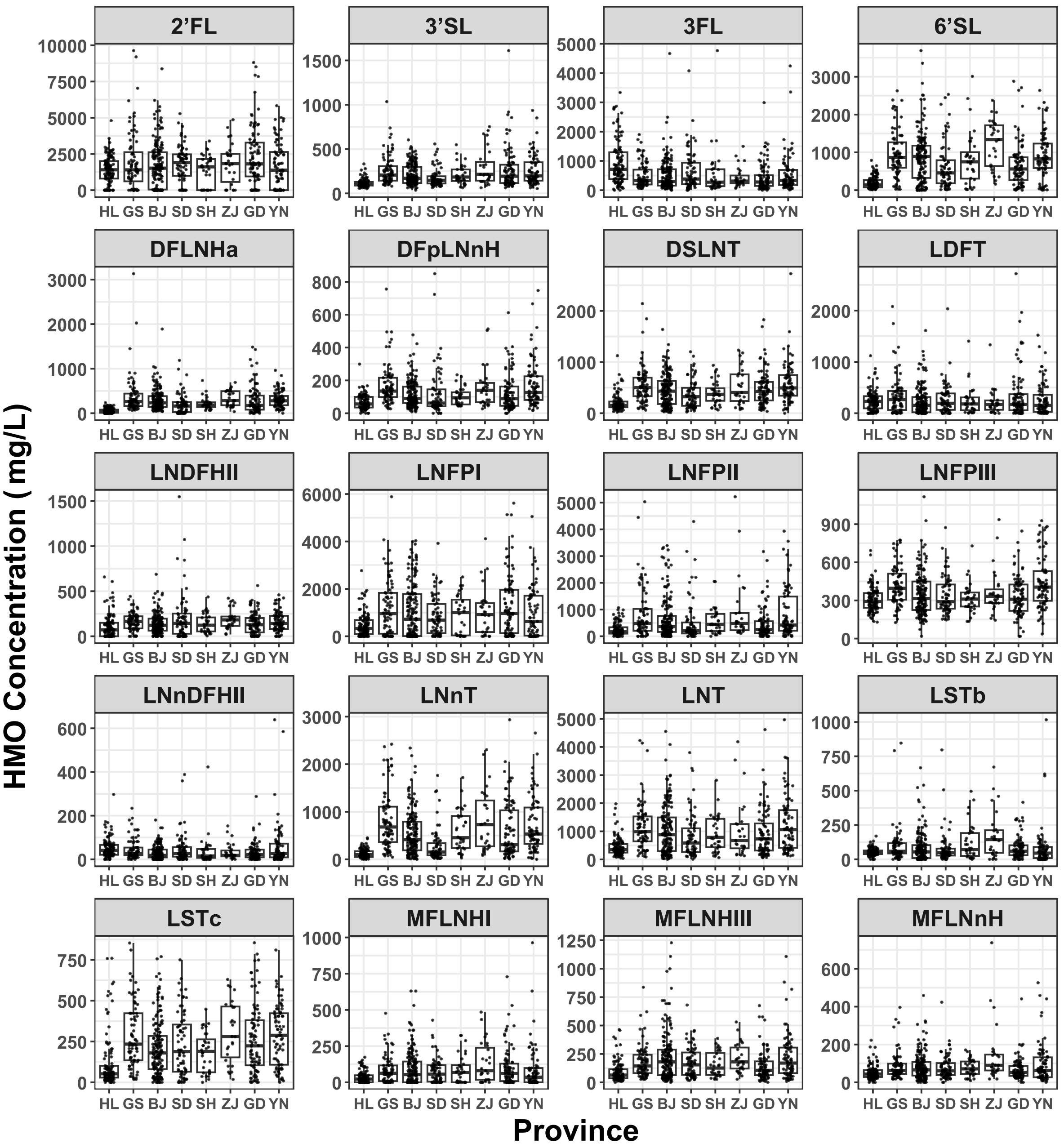

HL: Heilongjiang, GS: Gansu, BJ: Beijing, SD: Shandong, SH: Shanghai, ZJ: Zhejiang, GD: Guangdong, YN: Yunnan,

Supplementary Figure 2 HMO concentrations (mg/L) across provinces and municipalities

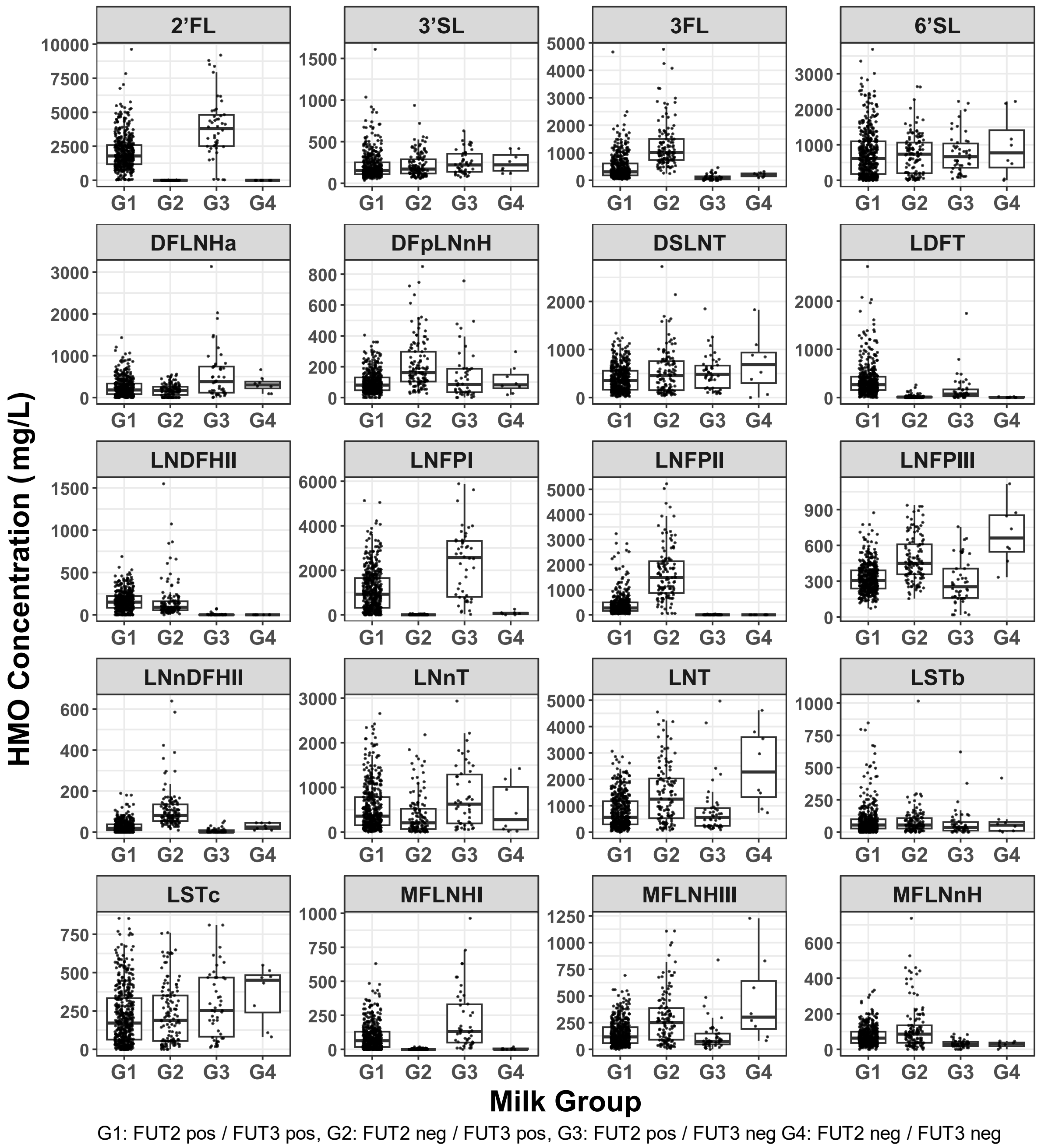

Supplementary Figure 3 HMO concentrations according to milk group. Boxplots show the median, interquartile ranges and the 95% confidence intervals. Individual data points are overlaid on each boxplot. FUT, fucosyltransferase; pos, positive; neg, negative.

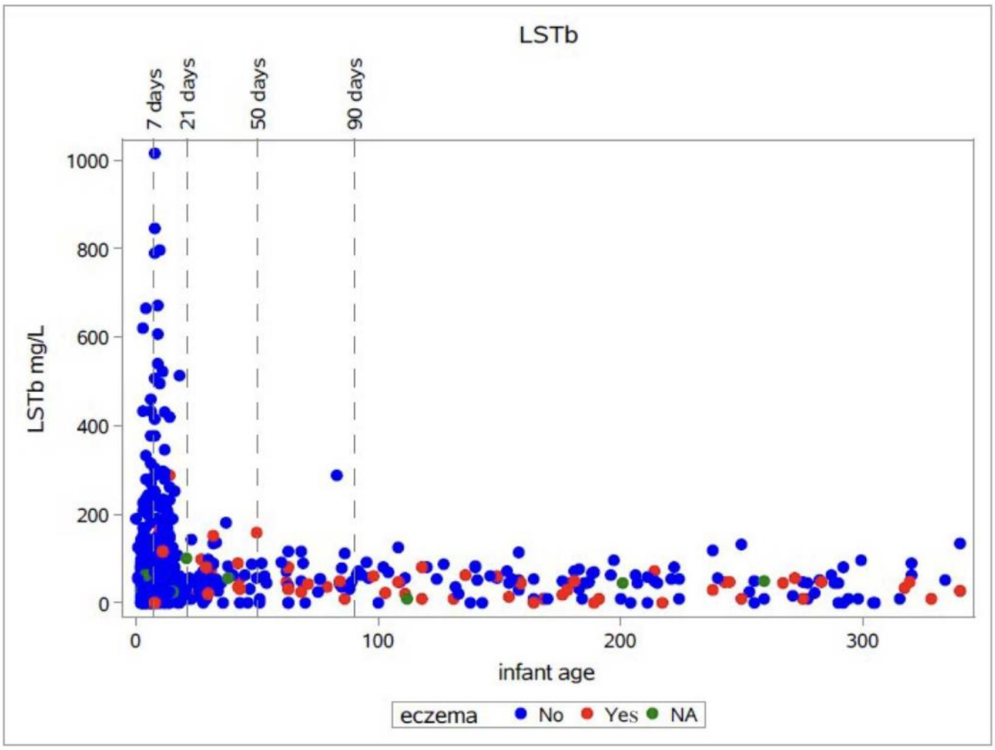

Supplementary Figure 4 LSTb concentrations (mg/L) and infant age (days) with eczema status
